# Memory CD8^+^ T Cells: Orchestrators and Key Players of Innate Immunity?

**DOI:** 10.1371/journal.ppat.1005722

**Published:** 2016-09-01

**Authors:** Grégoire Lauvau, Stanislas Goriely

**Affiliations:** 1 Albert Einstein College of Medicine, Department of Microbiology and Immunology, Bronx, New York, United States of America; 2 WELBIO and Institute for Medical Immunology, Université Libre de Bruxelles, Brussels, Belgium; University of Utah, UNITED STATES

## Abstract

Over the past decades, the dichotomy between innate and adaptive immune responses has largely dominated our understanding of immunology. Upon primary encounter with microbial pathogens, differentiation of adaptive immune cells into functional effectors usually takes several days or even longer, making them contribute to host protection only late during primary infection. However, once generated, antigen-experienced T lymphocytes can persist in the organism and constitute a pool of memory cells that mediate fast and effective protection to a recall infection with the same microbial pathogen. Herein, we challenge this classical paradigm by highlighting the “innate nature” of memory CD8^+^ T cells. First, within the thymus or in the periphery, naïve CD8^+^ T cells may acquire phenotypic and functional characteristics of memory CD8^+^ T cells independently of challenge with foreign antigens. Second, both the “unconventional” and the “conventional” memory cells can rapidly express protective effector functions in response to sets of inflammatory cytokines and chemokines signals, independent of cognate antigen triggering. Third, memory CD8^+^ T cells can act by orchestrating the recruitment, activation, and licensing of innate cells, leading to broad antimicrobial states. Thus, collectively, memory CD8^+^ T cells may represent important actors of innate immune defenses.

## Introduction

The dichotomy between fast, responsive innate immune cells of broad specificity and highly specific but slowly reacting adaptive immune cells has dominated the field of immunology in the last decades. In this view, innate immune responses provide early defense against invading pathogens and play an essential role in triggering and driving the acquired immune system to respond effectively to infection through the tailored expression of key mediators such as interleukin (IL)-12, type I interferons, and related cytokines by dendritic cell subpopulations [1]. In this context, naïve CD8 T cells that encounter their cognate antigen in lymphoid organs undergo expansion and activation. In a matter of days, they acquire expression of effector functions, such as interferon gamma (IFNγ), tumor necrosis factor (TNF), granzyme B, and perforin, that altogether contribute to pathogen clearance. While the majority of primed T cells undergo terminal differentiation into effector cells and ultimately die, a few percent will form long-lived memory after the infection is cleared [2,3]. Such memory cells are epigenetically programmed for more rapid and effective response upon re-stimulation with antigen [4]. Herein, we discuss why memory CD8 T cells should be considered as an important component of the early immune responses against invading pathogens and how their function is intimatly linked to that of innate immune cells.

## Differentiation into Memory CD8 T Cells in the Absence of Foreign Antigenic Exposure

**Fig 1:**
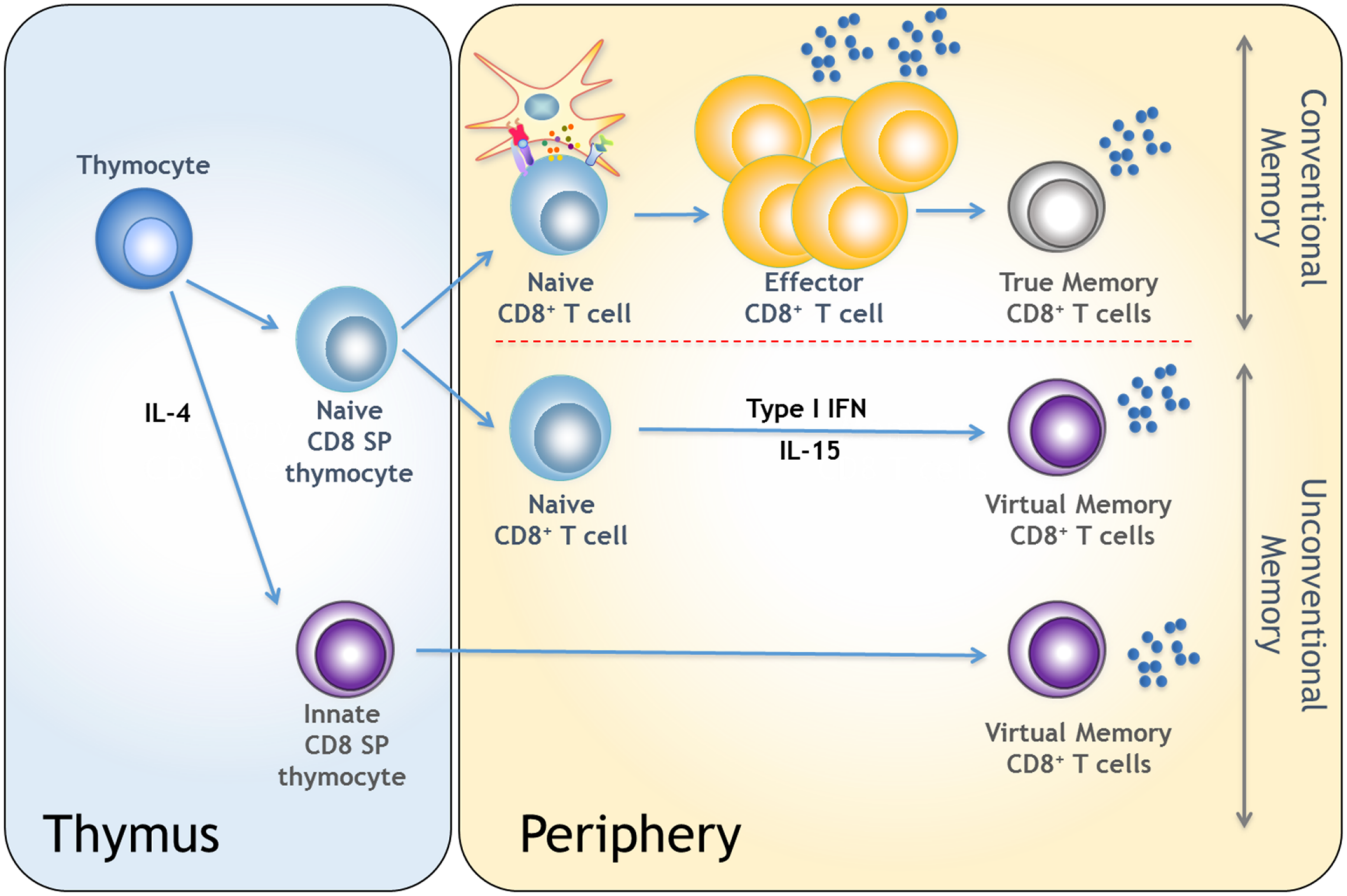
Pathway of conventional and unconventional CD8 T cell memory differentiation. Naïve CD8 T cells undergoing cognate antigen recognition in the context of an infection or an immunization differentiate into effector cells and form “true” antigen-experienced memory cells or "conventional memory." Under physiological conditions, naïve CD8 T cells may also acquire a memory phenotype in the absence of non-self cognate antigenic stimulation. This may occur in the thymus or in the periphery under the control of cytokines such as IL-4, IL-15, and type I IFN and give rise to “virtual memory” or "innate/memory-like" CD8 T cells.

Several unconventional pathways may lead to the formation of memory-like CD8 T cells (reviewed in [5,6]). It has long been known that naïve CD8 T cells in lymphopenic environment undergo conversion to memory phenotype CD8 T cells independent of foreign antigen exposure and in response to homeostatic cytokines [7]. Similar processes have more recently been extended to memory cells under physiological conditions in immunocompetent hosts (Fig 1). First, naïve CD8 SP thymocytes may already acquire a memory phenotype in the thymus under the influence of local IL-4 production [8]. The transcriptional networks involved in this unconventional differentiation process remain poorly understood, yet Eomesodermin (Eomes), an important T cell T-box transcription factor, appears to play a central role in driving these cells to acquire a phenotypic and functional memory phenotype [9,10]. Because they resemble other innate T cells such as invariant Natural Killer T (NKT) or γδ T cells as far as their activated/memory phenotype and their ability to rapidly produce cytokines, they were referred to as “innate” or “memory-like” CD8^+^ T cells [6]. Second, conversion of naïve CD8 T cells into memory-like cells without classical antigen-mediated differentiation also occurs in the periphery and accounts for the accumulation of memory cells upon ageing [11–13]. These cells, referred to as “virtual memory” CD8 T cells, display a classical “central memory” phenotype (CD44^+^CD62L^+^CD122^+^Bcl2^hi^). Their development also requires high expression of Eomes that controls CD122 expression—the transducing IL-15 receptor beta chain—and responsiveness to IL-15 trans presentation by CD8α^+^ dendritic cells [14]. Type I IFNs, produced under homeostatic conditions or during infections, drive Eomes expression and promote the development and expansion of memory-like CD8^+^ T cells [15]. Recently, Eomes^hi^ CD45RA^+^KIR^+^NKG2A^+^ “innate/memory-like” CD8^+^ T cells were also identified in human adult and cord blood samples [16,17]. As for their mouse counterpart, these cells were shown to traffic to the liver and to accumulate in older individuals [18]. Hence, a significant proportion—in fact, the majority in old mice—of the memory pool within secondary lymphoid organs represents cells that have never encountered their cognate antigen but are already primed to express rapid effector function [19]. Upon T cell receptor triggering, these cells respond faster and better than naïve CD8^+^ T cells of same antigenic specificity, yet they remain less effective than conventional memory CD8^+^ T cells, at least for proliferation and cytolysis [20]. Recent evidence also suggest that innate-like memory CD8 T cells may represent an important early line of defense against chronic viral infections [21]. These observations further blur the distinction between cells of the innate and the adaptive immune systems.

## Conventional and Non-Conventional Memory T Cells Exert Innate-Like Functions

**Fig 2:**
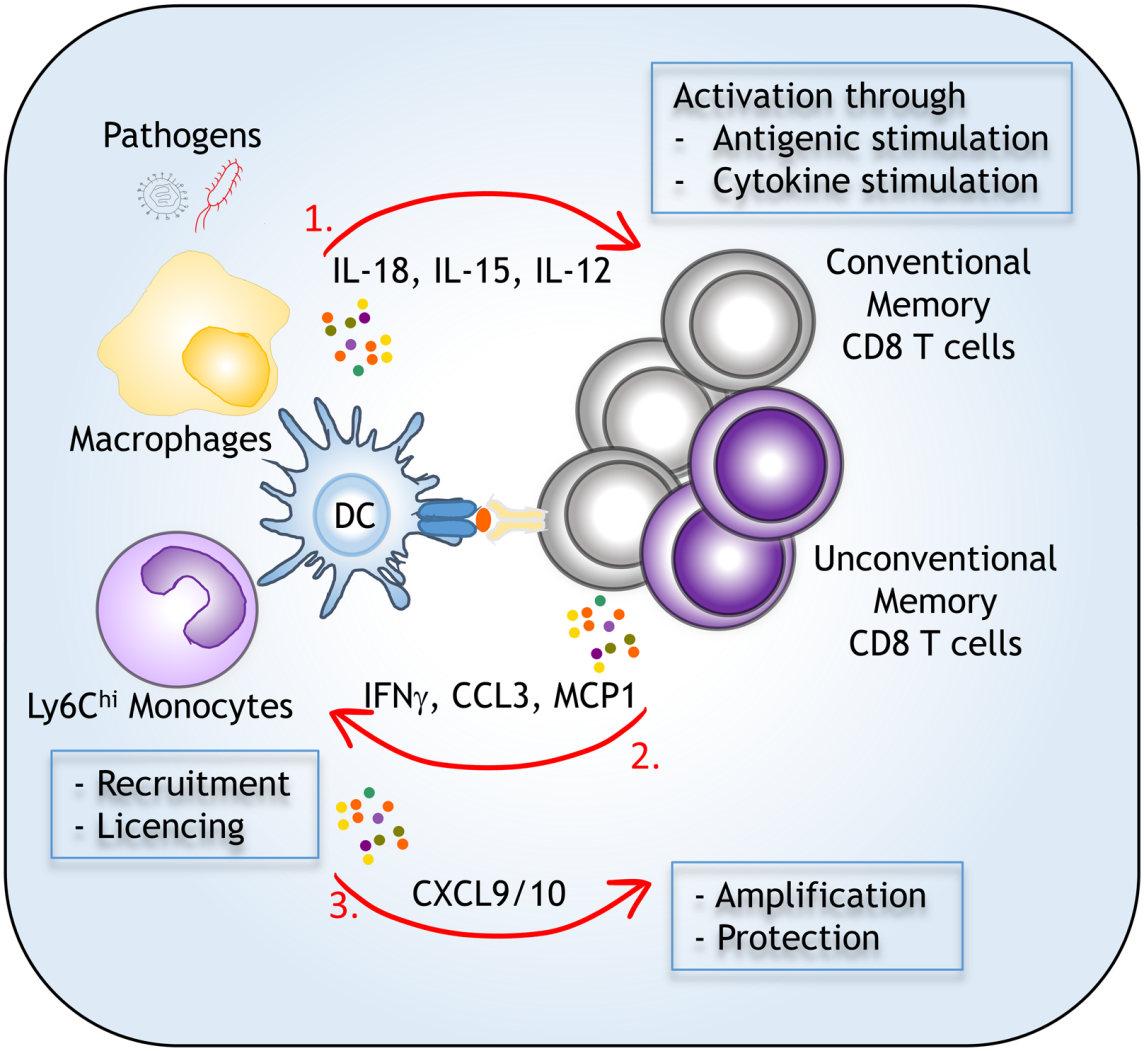
Mechanisms of memory CD8 T reactivation and orchestration of protective immune responses. Upon contact with microbial pathogen, different myeloid subpopulations may rapidly activate memory CD8 T cells through cytokinic and antigenic signals (Phase 1). In turn, memory CD8 T cells produce various cytokines and chemokines (IFNγ, CCL3) that allow initial recruitment and licencing of innate immune cells (Phase 2). Myeloid cells further amplify recruitment (CXCL9/10) of more memory T and innate effectors cells leading to pathogen containement and protective immunity (Phase 3).

Amongst the major functional characteristics acquired when naïve CD8 T cells differentiate into conventional or unconventional memory CD8 T cells is their capacity to "sense" and respond to inflammatory cytokines. Such features were previously thought to be restricted to NK cells and other innate lymphoid subpopulations such as NKT or γδ T cells. Conventional αβ memory CD8 T cells are able to rapidly produce important quantities of IFNγ in the spleen and the draining lymph nodes (dLNs) of infected mice in response to homologous or even heterologous challenge infections and independent of cognate antigen recognition (Fig 2) [22–25]. Unconventional memory CD8 T cells share the same property [8,19]. In dLNs, memory CD8 T cells are spatially prepositioned close to lymphatic sinus-lining sentinel macrophages [26]; therefore, they rapidly and efficiently receive inflammasome-generated IL-18 from pathogen-sensing phagocytes [27]. Recruitment of central memory CD8 T cells to the dLN macrophages involves CXCL10 secreted by the macrophages in response to pathogen sensing and autocrine type I IFN [28]. Likewise, IL-18, IL-15, and CXCL9 produced by CD8α^+^ DCs (including XCR1^+^ DCs) and inflammatory Ly6C^hi^ monocytes promote both rapid mobilization and expression of effector functions by conventional memory CD8^+^ T cells [23,25,29]. Such cytokine-driven activation of memory CD8^+^ T cells contributes to innate responses and protection in vivo. Along the same line, NKG2D-mediated killing by memory CD8^+^ T cells was also shown to participate in the early control of pathogen replication [30]. Altogether, this body of work challenges the view that antigenic recognition and clonal expansion are necessarily required for memory CD8 T cells to exert protective effector functions. However, achieving full protection and sterilizing immunity against microbial pathogen infections requires the presence of cognate antigen.

## Memory CD8 T Cells Orchestrate Innate Immune Responses

It is widely assumed that protection conferred by memory CD8 T cells is largely dependent on direct perforin- and Fas-mediated cytolysis of pathogen-infected cells [31]. While the role of non-cytolytic mechanisms in the control of microbial pathogen infections by memory CD8^+^ T cells, such as production of effector cytokines like IFNγ, was appreciated long ago [32], the relative importance of such indirect mechanisms has not been thoroughly investigated. Early reports using adoptively transferred effector CD8 T cells genetically lacking cytolytic mechanisms (perforin knockout) and IFNγ or even TNF in the *Listeria monocytogenes* infection model [33,34] and in models of transplanted metastatic tumors [35] suggested that non-cytolytic mechanisms may contribute significantly to microbial pathogen and tumor clearance. Recent evidence reveal that upon both antigen and/or cytokine-driven reactivation, systemic (from the circulating and the secondary lymphoid organ [SLO] pool) and tissue-resident (from the mucosa) memory CD8 T cells orchestrate subsequent innate immune cell responses. Through rapid IFNγ production, memory T cells can promote recruitment (via CXCL9 and other chemokines), activation, and licensing of multiple subsets of innate myeloid and lymphoid cells, leading to a "broad antimicrobial state" and subsequent bacterial and viral clearance (Fig 2) [36–38]. IFNγ together with additional mechanisms implicating recruiting chemokines were also reported to be essential in memory CD8 T cell-mediated protection. CCL3 in particular participates to the recruitment and activation of inflammatory Ly6C^hi^ monocytes and neutrophils and, together with direct IFNγ signaling to these phagocytes [36], leads to increased production of TNF and microbicidal reactive oxygen species (ROS) promoting antimicrobial autophagy [39]. These events allow fast control of pathogen growth in vivo and can account for host protection. Skin commensal-specific memory CD8 T cells may also promote innate cell barrier immunity through IL-17 production and induction of antimicrobial peptides by epithelial cells [40].

## Conclusions and Perspectives

Collectively, these data shed novel light on mechanisms involved in memory CD8 T cell-mediated protection reactivation and innate-like characteristics. They also reveal the importance of non-cytolytic as well as antigen-independent mechanisms in the protection of vaccinated hosts and should help us revise our current understanding of immune responses in general and how innate and adaptive immune cells work together. The extent of antigen-independent protection conferred by conventional or unconventional memory CD8 T cells has been quite thoroughly evaluated by several groups in different experimental systems, and data establish a clear contribution to host protection [18,23–25,41]. Yet, a very important, still open question relates to antigen-dependent non-cytolytic versus cytokinic mechanisms of host protection, which will likely depend on the nature of each infection. We focused this view on mechanisms beneficial to the host. However, in some settings cytokine-mediated activation of T cells can lead to immunopathology. NKG2D-mediated killing is one such example [42]. In obesity-related inflammation, MCP-1 derived from CD8 T cells may promote recruitment and deleterious activation of macrophages [43]. Hence, the “innate function” of memory CD8 T cells needs further evaluation in the context of autoimmune and inflammatory disorders.
